# Cytomegalovirus infection associated with smaller cerebellum in severe mental illness

**DOI:** 10.1192/j.eurpsy.2023.297

**Published:** 2023-07-19

**Authors:** D. Andreou, K. N. Jørgensen, S. Nerland, O. A. Andreassen, R. H. Yolken, I. Agartz

**Affiliations:** 1 Department of Psychiatric Research, Diakonhjemmet Hospital; 2 Norwegian Centre for Mental Disorders Research (NORMENT), Institute of Clinical Medicine, University of Oslo, Oslo, Norway; 3 Centre for Psychiatry Research, Department of Clinical Neuroscience, Karolinska Institutet & Stockholm Health Care Services, Stockholm County Council, Stockholm, Sweden; 4 Department of Psychiatry, Telemark Hospital, Skien; 5 Norwegian Centre for Mental Disorders Research (NORMENT), Division of Mental Health and Addiction, University of Oslo, Oslo, Norway; 6 Stanley Division of Developmental Neurovirology, Department of Pediatrics, Johns Hopkins University School of Medicine, Baltimore, MD, United States

## Abstract

**Introduction:**

Postnatal cytomegalovirus (CMV) infection of immunocompetent hosts is usually inapparent but typically results in lifelong latency. Congenital CMV infections as well as CMV infections in patients with immunodeficiencies have been linked to major cerebellar pathology. Patients with severe mental illness have been repeatedly found to have smaller cerebellum, and they may be particularly susceptible to CMV infections. Finally, both animal and human studies have shown a differential male and female immune response to CMV.

**Objectives:**

We evaluated whole cerebellar grey matter volumes (CGMV) in CMV immunoglobulin G (IgG) seropositive (CMV+) and seronegative (CMV-) patients with severe mental illness and healthy controls (HC). We hypothesized that CMV seropositivity, reflecting previous infection and current latency, is associated with smaller CGMV in patients but not in HC, and that such a putative association may be sex-dependent.

**Methods:**

We included 529 adult patients with severe mental illness (CMV+ 57%, women 48%), i.e., 324 patients with schizophrenia spectrum disorders and 205 patients with bipolar spectrum disorders, and 494 HC (CMV+ 56%, women 45%). MRI scans were obtained with a 1.5T Siemens scanner (n=596) and two 3.0T General Electric scanners (n=427), and processed with FreeSurfer v6.0. Circulating CMV IgG concentrations were measured with immunoassays. In age-, scanner- and estimated total intracranial volume-adjusted analyses of covariance (ANCOVAs), we investigated main and interaction effects of CMV status and sex on CGMV in patients and HC.

**Results:**

CMV+ patients had smaller CGMV than CMV- patients (p=0.042). There was no CGMV difference between CMV+ and CMV- HC (p=0.858). The adjusted CGMV means in CMV+ patients and CMV- patients were 115078 mm^3^ and 116725 mm^3^, respectively (p=0.042); the adjusted CGMV means in CMV+ and CMV- HC were 117980 mm^3^ and 117840 mm^3^, respectively (p=0.858) (Image). Among patients, a trend towards CMV-by-sex interaction (p=0.073) was found. Post-hoc analyses showed a significant CMV-CGMV association in the female patient group (p=0.005), with no association among male patients (p=0.840).

**Image:**

**Figure:**
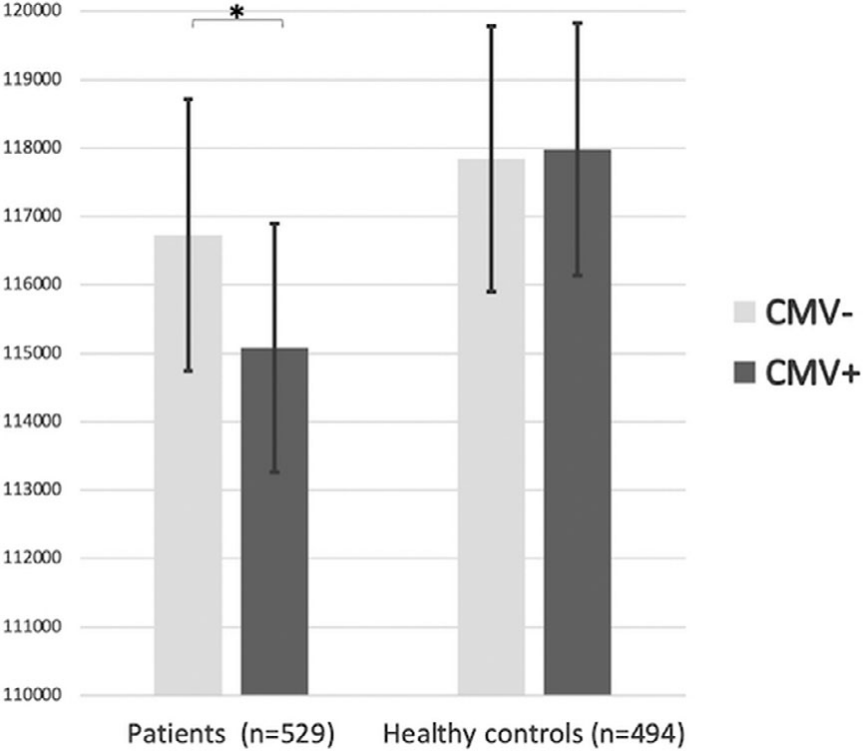

**Conclusions:**

CMV IgG seropositivity is associated with smaller cerebellum in severe mental illness, an effect driven by the female patients, but not among HC. This may indicate a CMV-related deleterious impact on cerebellum restricted to patients.

**Disclosure of Interest:**

None Declared

